# PR-39, a porcine host defence peptide, is prominent in mucosa and lymphatic tissue of the respiratory tract in healthy pigs and pigs infected with *actinobacillus pleuropneumoniae*

**DOI:** 10.1186/1756-0500-5-539

**Published:** 2012-09-28

**Authors:** Isabel Hennig-Pauka, Rüdiger Koch, Doris Hoeltig, Gerald-F Gerlach, Karl-Heinz Waldmann, Frank Blecha, Carsten Brauer, Hagen Gasse

**Affiliations:** 1 Clinic for Swine, University of Veterinary Medicine, Vienna, Austria; 2 Institute of Anatomy, University of Veterinary Medicine Hannover, Foundation, Hannover, Germany; 3 Clinic for Swine and Small Ruminants, University of Veterinary Medicine Hannover, Foundation, Hannover, Germany; 4 Institute for Innovative Veterinary Diagnostics, Hannover, Germany; 5 Department of Anatomy and Physiology, College of Veterinary Medicine, Kansas State University, Manhattan, Kansas, USA

**Keywords:** Swine, Innate immunity, Cathelicidin, Antibacterial peptide, Pleuropneumonia, Respiratory tract

## Abstract

**Background:**

Host defence peptides are important components of mammalian innate immunity. We have previously shown that PR-39, a cathelicidin host defence peptide, is an important factor in porcine innate immune mechanisms as a first line of defence after infection with *Actinobacillus pleuropneumoniae*. PR-39 interacts with bacterial and mammalian cells and is involved in a variety of processes such as killing of bacteria and promotion of wound repair. In bronchoalveolar lavage fluid of infected pigs PR-39 concentrations are elevated during the chronic but not during the acute stage of infection when polymorphonuclear neutrophils (known as the major source of PR-39) are highly increased. Thus it was assumed, that the real impact of PR-39 during infection might not be reflected by its concentration in bronchoalveolar lavage fluid.

**Results:**

Using immunohistochemistry this study demonstrates the actual distribution of PR-39 in tissue of the upper and lower respiratory tract of healthy pigs, and of pigs during the acute and chronic stage of experimental infection with *Actinobacillus pleuropneumoniae*.

During the acute stage of infection PR-39 accumulated adjacent to blood vessels and within bronchi. Immune reactions were mainly localized in the cytoplasm of cells with morphological characteristics of polymorphonuclear neutrophils as well as in extracellular fluids. During the chronic stage of infection pigs lacked clinical signs and lung alterations were characterized by reparation and remodelling processes such as tissue sequestration and fibroblastic pleuritis with a high-grade accumulation of small PR-39-positive cells resembling polymorphonuclear neutrophils. In healthy pigs, PR-39 was homogenously expressed in large single cells within the alveoli resembling alveolar macrophages or type 2 pneumocytes. PR-39 was found in all tissue samples of the upper respiratory tract in healthy and diseased pigs. Within the tracheobronchial lymph nodes, PR-39 dominated in the cytoplasm and nuclei of large cells resembling antigen-presenting cells located in the periphery of secondary follicles.

**Conclusions:**

These immunohistochemical findings indicate that, in addition to polymorphonuclear neutrophils, other cells are involved in the expression, storage, or uptake of PR-39. The presence of PR-39 in healthy lung tissue showed that this antibacterial peptide might be important for the maintenance of health.

## Background

Multifactorial respiratory diseases are an important cause of economic losses in the swine industry [1,2]. Microbial colonization and invasion, and abiotic factors, such as climate, social stress and nutrition, as well as host factors like innate and adaptive immune responses, are decisive for the outcome of disease [3,4]. Knowledge of the distribution of key factors of the innate immune system is important not only for examining host-pathogen-interactions but also for assessing vaccination strategies with respect to a successful stimulation of the innate immune system of the respiratory tract. Pigs have several host defence peptides with ß-defensins and cathelicidins as the most important ones. With eleven porcine cathelicidins the pig is endowed with the most diverse family of cathelicidins compared to other species [5]. Linear proline-rich cathelicidins such as PR-39 form a type II poly-L-proline helix facilitating an interaction with bacterial and mammalian cells without translocation across biological membranes. The high proline content of this peptide may explain its stability in highly proteolytical microenvironments (e.g. inflammation sites, mucosal surfaces) [5]. PR-39 was initially characterized as an antibacterial factor in the intestine of pigs [6,7]. Increased serum PR-39 levels have been found after infection with *Salmonella choleraesuis *[5,8]. PR-39 is also known as a multifunctional peptide involved in a variety of processes, including promotion of wound repair, induction of angiogenesis, regulation of apoptosis, neutrophil chemotaxis and inhibition of NADPH oxidase activity [5,7]. PR-39 is released in wound fluid by recruited polymorphonuclear neutrophils (PMNs) and serves as a signalling factor for the induction of syndecan [9].

For investigating host-pathogen interactions during pneumonia, standardised infection models are indispensable. Infection with *Actinobacillus pleuropneumoniae* (*A.pp*.), a highly pathogenic bacterial porcine respiratory tract pathogen, is well established for examining porcine host reactions as well as changes in bacterial virulence factor expression during infection [10,11]. The chronic stage of *A.pp.* infection is characterised by reparative processes like sequestered necroses and persistent pleural adhesions [12,13]. The concentration of PR-39 was significantly increased in bronchoalveolar lavage fluid and correlated to PMNs as well as to the lung lesion score in this chronic stage of *A.pp*. infection. It was assumed that the neutrophilic origin of PR-39 as well as a hypothetical involvement in tissue repair processes were the reason for this observation [9,14]. No correlation of PR-39 concentrations with cellular parameters was found in the acute stage of *A.pp*. infection in bronchoalveolar lavage fluid although PMNs were highly increased [14]. It was anticipated that the real impact of PR-39 during different stages of infection might not be reflected by its concentration in bronchoalveolar lavage fluid.

Our research hypothesis was that not only PMNs but also other cells were involved in the expression, storage, or uptake of PR-39. Furthermore, it was assumed that PR-39 positive cells can only be detected in tissue of diseased pigs.

## Methods

### Experimental infection and clinical investigations

German Landrace pigs of both sexes used in this study were 6 to 7 weeks old and originated from a single herd routinely monitored and free from endo- and ectoparasites, toxigenic *Pasteurella multocida*, *A.pp*. and Porcine Reproductive and Respiratory Syndrome Virus. In previous studies, the German Landrace, as one of the most common purebred pigs in Germany has been found to be highly susceptible towards *A.pp.* infection and has proven itself to be suitable for reliable and repeatable challenge experiments [11]. Immunohistological examination of tissue samples was performed on three healthy mock-infected pigs and eight pigs infected with *A. pp*.serotype 7 in the acute (2) and chronic (6) stage of infection (Table 1). Groups were housed in separate isolation units under standard conditions and cared for in accordance with the principles outlined by the European Convention for the Protection of Vertebrate Animals used for Experimental and Other Scientific Purposes (European Treaty Series, nos. 123 http://conventions.coe.int/treaty/EN/treaties/html/123.htm and 170 http://conventions.coe.int/treaty/EN/treaties/html/170.htm; approval number: 33-42502-05/ 941) and clinical signs were monitored daily. Aerosol infection was performed using the *A. pp.* serotype 7 strain AP76 diluted in 154 mM sodium chloride resulting in approximately 1 x 10^5^ colony forming units per millilitre suspension for nebulization as previously described [15,16]. 154 mM sterile sodium chloride was used for mock infection. The project was approved from the Lower Saxony State Office for Consumer Protection and Food Safety as the responsible regulatory authorities and in accordance with the requirements of the national animal welfare law fulfilling also international recognized guidelines (approval number: 33-42502-05/ 941).

In all pigs living until day 21 after infection, bacteriological examination and PCR analysis of altered and unaltered lung tissue were performed to determine persistent infection with *A.pp.* In addition, lung tissue samples of two mock infected control pigs (Table 1, pigs 2 and 3) were examined for colonizing bacterial species. Pigs were necropsied and lung lesions were analysed following the evaluation scheme proposed by Hannan et al. [17]. In brief, the size and position of lesions were mapped on a simplified lung chart representing the seven lung lobes, which are subdivided into triangles. A maximum possible score of 5 can be allotted to each lung lobe. The number of affected triangles in one lung lobe represents the pneumonic area, which is assessed as a fraction of 5 (resulting in a maximum score of 35 for the complete lung). Lung lesion scores and the findings of histological tissue examination are shown in Table 1.

**Table 1:** **Macroscopic and microscopic findings in lungs of mock-infected pigs and pigs infected with *Actinobacillus pleuropneumoniae***

**Pig**	**Infection**	**Day of necropsy after infection**	**Macroscopic lung lesion score **[17]	**Histological findings**
1	mock	21	0	Predominantly without pathological findings, small areas with slight interstitial pneumonia
2	mock	21	3.6	Predominantly without pathological findings, small areas with slight interstitial pneumonia
3	mock	21	0	Predominantly without pathological findings, small areas with slight interstitial pneumonia
4	*A.pp*. serotype 7 strain AP76	1	17.7	Severe purulent and fibrinous pneumonia, moderate interstitial pneumonia
5	*A.pp*. serotype 7 strain AP76	21	12.3	Severe catarrhal and purulent bronchopneumoniae with abscesses, slight interstitial pneumonia
6	*A.pp*. serotype 7 strain AP76	21	6.3	Severe purulent pneumonia with abscesses and sequesters with colliquative necrosis
7	*A.pp*. serotype 7 strain AP76	21	0	Predominantly without pathological findings, small areas with severe interstitial pneumonia
8	*A.pp*. serotype 7 strain AP76	21	4.4	Moderate catarrhal and purulent bronchopneumonia with abscesses, moderate interstitial pneumonia
9	*A.pp*. serotype 7 strain AP76	21	0	Most areas without pathological findings, small areas with severe interstitial pneumonia
10	*A.pp*. serotype 7 strain AP76	4	31.4	Severe purulent and necrotic bronchopneumonia, fibrinous tissue alterations
11	*A.pp*. serotype 7 strain AP76	21	6.4	Severe purulent pneumonia with fibrotic tissue alterations, moderate interstitial pneumonia

### Immunohistochemistry for PR-39

Tissue samples were collected following a standard protocol to ensure the comparability of equal localizations in different individuals. The head was separated from the body and cut paramedially in a longitudinal direction, and, subsequently, transversally at a level with the first premolar tooth to expose the nasal cavity. The nasal septum was removed. To follow the route of natural lung infection, tissue sampling was performed as follows: a disc-shaped transversal cut sample of the dorsal and ventral nasal concha was taken; subsequently, samples from the pharyngeal tonsil and trachea (approximately 50 mm cranial to the bifurcation) were excised. Samples were taken from the caudal lung lobes from unaltered and altered lung tissue well as from the tracheobronchial lymph nodes. Samples were fixed in Bouin’s fluid containing 0.9% picric acid, 9% formaldehyde and 5% glacial acetic acid for 48 h at room temperature and afterwards dehydrated in a graded series of ethanol before embedding through xylene in paraffin wax (Paraplast plus, Tyco Health Care). Paraffin sections (8 μm) were cut with a motor driven rotation microtome (Autocut, Reichert-Jung), deparaffinised in Histoclear (Life Science Int.) and hydrated through descending concentrations of ethanol. Routinely, histological sections were either stained with haematoxylin-eosin (HE, haemalaun after Delafield) or with Masson’s trichrome stain. For detecting PR-39, the sections were stained immunohistochemically with a monoclonal mouse anti-PR-39-antibody [8] in a dilution of 1:400 overnight after a thirty-minute incubation step with normal goat serum (BioGenex, San Ramon, USA). Subsequently, a secondary immunohistochemical detection system employing an indirect biotin-streptavidin amplified system was performed according to the manufacturer’s instructions (Super Sensitive™ Link-Label IHC Detection System, BioGenex, San Ramon, USA). In brief, tissue sections were incubated with a biotinylated anti-mouse immunglobulin and then with Horseradish Peroxidase-labelled streptavidin. The entire antibody-enzyme complex was made visible by the addition of a 3, 3’-diaminobenzidine (DAB) chromogen solution. Control sections were incubated without the first antibody. Sections were photographed with a Zeiss Photomicroscope II equipped with a digital camera (Olympus DP70).

## Results and discussion

### Development of porcine pleuropneumonia in infected animals

All infected pigs developed acute clinical signs such as an increase in body temperature, inappetence, lethargy and dyspnoea. Six pigs recovered within the first week and were assessed as clinically healthy before necropsy on day 21/22. Two pigs (Table 1: pigs 4 and 10) died during the acute stage of infection from severe pneumonia. Mock infected animals showed no clinical signs (Table 1). Fibrinous pleuritis and pneumonia as well as lung oedema and haemorrhage dominated in the first four days after infection, while fibroblastic pleuritis and lung tissue sequestration were found in four chronically infected pigs. Mock-infected pig 2 showed a slight catarrhalic purulent bronchopneumonia of the right cranial lung lobe characteristic for enzootic pneumonia. A histological examination revealed an interstitial pneumonia in nearly all pigs (Table 1). Whether interstitial pneumonia is a balanced reaction to commensal bacteria or due to other microorganisms belonging to the Porcine Respiratory Disease Complex, e.g. *Mycoplasma hyopneumoniae* and Porcine Circovirus 2, cannot be answered. In a recent study 50% of healthy Danish finishing pigs used as control animals for histopathological examination showed microscopic lung alterations [1].

*A.pp.* could be reisolated from the six infected pigs with lung alterations. Mock infected pigs stayed negative for *A.pp.;* Isolated bacterial species (*Bordetella bronchiseptica*, *Escherichia coli,* α-haemolytic streptococci) belonged to the spectrum of species which are isolated regularly from bronchoalveolar lavage fluid samples of clinically healthy and specific pathogen-free pigs. The presence of a normal flora of the upper respiratory tract is assumed [18].

### Demonstration of PR-39 in lung tissue of infected and non-infected pigs

**Figure 1:**
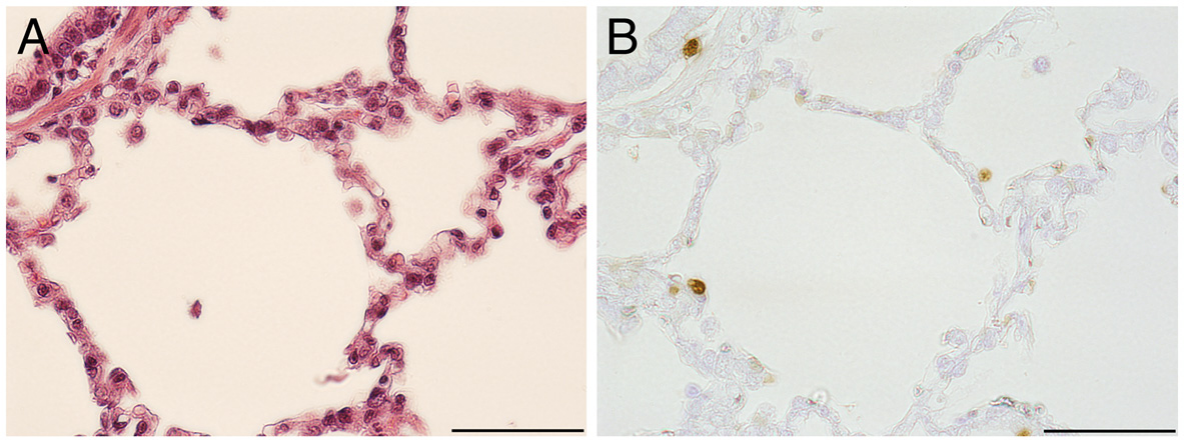
**Immunohistochemical demonstration of PR-39 positive cells in lung tissue of mock infected pig 3. A**) Semiserial haematoxylin-eosin stained histological section, bar 50 μm. **B**) PR-39 positive cells are distributed evenly within the lung tissue. Single large positive cells in the walls of every alveolus, bar 50 μm.

Positive staining reactions for PR-39 were obtained from all organ tissue samples (lung, upper airways, lymphatic) of all animals examined. However, striking differences between healthy and diseased pigs were only found in the tissue samples from the lung. In unaltered lung tissue of infected and uninfected pigs PR-39-positive cells were evenly distributed in the pulmonary tissue and were regarded as alveolar pneumocytes type 2 or alveolar macrophages due to their location within the alveolar wall (1–2 cells/alveolus, Figure 1). The presence of PR-39 in healthy lung tissue might be one important factor for the maintenance of health as a dynamically balanced process under the influence of microorganisms, environmental factors and host reactions.

**Figure 2:**
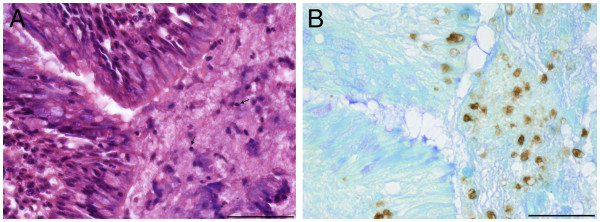
**PR-39 positive cells in lung tissue of pig 11 on day 21 after infection. A**) Semiserial haematoxylin-eosin stained histological section with necrotic areas characterized by pyknosis (arrows) and amorphous cellular debris, bar 50 μm. **B**) PR-39 positive cells occur within necrotic areas, bar 50 μm.

**Figure 3:**
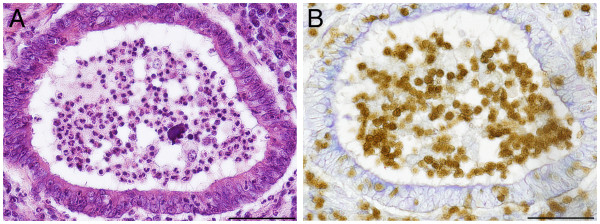
**PR-39 positive cells in the lumen of bronchi of pig 5 on day 21 after infection. A**) Semiserial haematoxylin-eosin stained histological section. The morphology of cells in the lumen of bronchus resemble PMNs, bar 50 μm. **B**) PR-39 localization in the cytoplasm and nucleus of small cells in the lumen of a bronchus, bar 50 μm.

During acute infection positive cells accumulated at the pleura, around blood vessels and bronchi and in bronchial secretions which are dominated by PMNs. Surprisingly, no correlation between the concentration of free PR-39 and cellular parameters in bronchoalveolar lavage fluid were found in previous studies in this acute stage of infection. In the chronic stage of infection PR-39-positive small cells accumulated in demarcation zones to necrotic areas and also occur within necrotic areas (Figure 2) as well as in the bronchial spaces (Figure 3). The immunopositive signal was located in the nucleus and in the cytoplasm.

Concentration of PR-39 in epithelial lining fluid (ELF) covering the airways is deduced to be approximately 200 nM in diseased pigs [14]. *In vitro*, no bactericidal activity of PR-39 against *A.pp.* has been previously observed , although bactericidal activity of PR-39 is well documented against other gram-negative bacteria with lower concentrations [19-21]. Resistance of *A.pp.* to PR-39 is in accordance with the *in vivo* observation that *A.pp.* persists for extended periods of time on the respiratory epithelium of pigs [22,23]. Most probably, PR-39 plays a synergistic role with other antimicrobial factors in the porcine respiratory tract, as has been shown for other antimicrobial peptides at sub-inhibitory concentrations [24,25]. The multiplicity of antimicrobial factors in the ELF and their synergistic effects might reduce the time available for bacteria to develop a more virulent phenotype. Furthermore, the combination of antimicrobials might prevent emergence of strains resistant to endogenous antimicrobials [25].

### Demonstration of PR-39 in the upper respiratory tract and lymphatic tissue

The PR-39 staining pattern of nasal and tracheal mucosa, pharyngeal tonsil and tracheobronchial lymphnodes was similar in infected and non-infected pigs. This may reflect the fact that the mucosa of the upper respiratory tract, which is known to be colonized by a commensal microbial flora, is continuously influenced by environmental as well as internal factors and demands an innate system of constitutively produced stabilizing and homeostatic substances. Porcine beta-defensin as the other prominent porcine host defence peptide was identified previously in epithelial cells of the porcine tongue and buccal mucosa in healthy pigs. The intraepithelial defensin shows synergistic bactericidal activity with PR-39 from inflammatory neutrophils and is supposed to enhance microbicidal defence in porcine mucosal surfaces [26].

**Figure 4:**
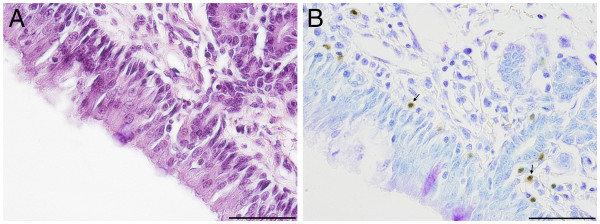
**Immunohistochemical demonstration of PR-39 in nasal mucosa of mock infected pig 3. A**) Semiserial haematoxylin-eosin stained histological section, bar 50 μm. **B**) PR-39 is localized within the cytoplasm of small positive cells with segmented nuclei resembling PMNs (arrows) in subepithelial tissue, bar 50 μm.

**Figure 5:**
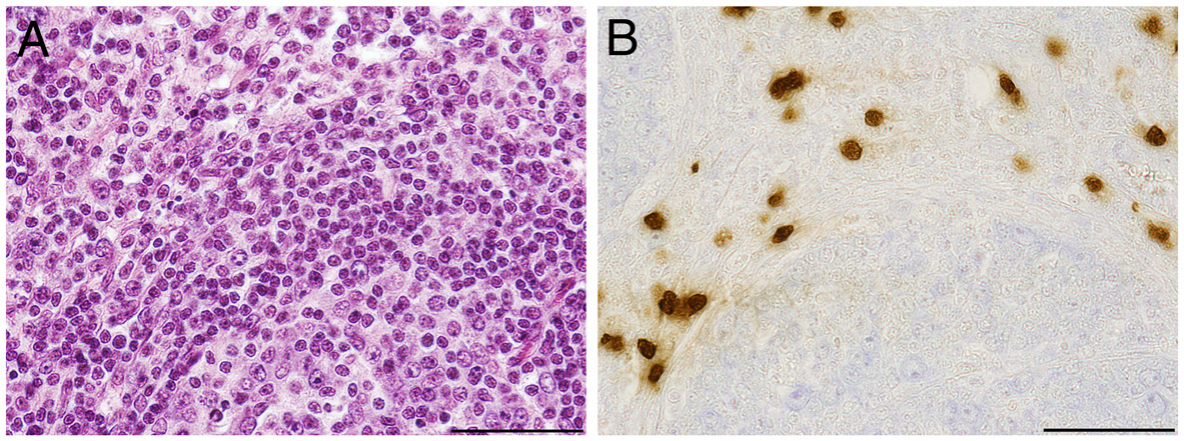
**Immunohistochemical demonstration of PR-39 positive cells in the tracheobronchial lymph node of pig 6 on day 21 after infection. A**) Semiserial haematoxylin-eosin stained histological section, bar 50 μm. **B**) Large PR-39 positive cells are localized within the cortical and medullar parenchyma, to a lesser extent in intermediary and subcapsular sinus, but not within the secondary nodules, bar 50 μm.

An accumulation of PR-39 positive cells was observed around epithelial lesions, which were more frequent in infected animals. In general, according to morphological characteristics, different cell types appeared immunopositive, namely i) small cells with positive cytoplasms and positive segmented nuclei and ii) large cells with large, positive nuclei. In the nasal mucosa small positive cells predominated. They were located subepithelially in the lamina propria (Figure 4). In the mucosa of the pharyngeal tonsil, positive cells accumulated around lymphatic follicules partially reaching the mucosal surface. Single large positive cells as well as small cells were found subepithelial in the tracheal mucosa of healthy and diseased pigs. Within lymphnodes PR-39-positve cells accumulated in the cortical and medullar parenchyma, to a lesser extent in intermediary and subcapsular sinuses, but not within the secondary nodules. These regions are known to comprise lymphocytes, plasma cells, macrophages, and neutrophils. According to their size and shape, PR-39 positive cells resembled antigen-presenting cells with large bright nuclei (Figure 5). Unfortunately most antibodies for cell markers in swine cannot be used for immunohistochemistry in paraffin-embedded tissue. Using SWC3/172a, as the first established porcine myelomonocytic marker, a distinction between granulocytes, monocytes, macrophages and plasmacytoic dendritic cells is not possible [27]. In future studies staining protocols for paraffin-embedded tissue have to be elaborated to test available monoclonal antibodies against cell markers.

## Conclusion

The detection of PR-39 in cells of different shape, size, and location indicates that this peptide is not restricted to only one type of specific cell, but rather to various cell types assigned to different functions within the network of innate immune mechanisms. The morphology of cells positive for PR-39 suggests PMNs. However, large immune cells are also implied. A further characterisation of the cells by double staining for PR-39 and swine myelomonocytic differentiation antigens would be necessary for a more precise identification of the positive cells.

The hypothesis that PR-39 is only detectable in diseased pigs can be rejected. The presence of PR-39 in tissue of the upper and lower respiratory tract of healthy pigs shows that this antibacterial peptide might be important for maintaining health under the influence of microorganisms and environmental factors.

## Competing interests

The authors declare that they have no competing interests.

## Authors’ contributions

IHP participated in the design of the study, was involved in clinical examinations and necropsies, evaluated the histological sections and drafted the manuscript. RK participated in immunohistochemistry and carried out the photography of sections. DH participated in the clinical studies. GFG organized the clinical studies and carried out experimental infections. KHW participated in the design and the coordination of the studies. FB developed the monocloncal antibody used for immunohistochemistry and helped to draft the manuscript. CB carried out the necropsies and calculated the lung lesion scores. HG conceived the study, participated in necropsies, interpreted immunohistochemical findings and helped to draft the manuscript. All authors read and approved the final manuscript.
